# Traditional Chinese Medicine Interventions in the Rehabilitation of Cognitive and Motor Function in Patients With Stroke: An Overview and Evidence Map

**DOI:** 10.3389/fneur.2022.885095

**Published:** 2022-05-17

**Authors:** Tae-Young Choi, Ji Hee Jun, Hye Won Lee, Jong-Min Yun, Min Cheol Joo, Myeong Soo Lee

**Affiliations:** ^1^KM Science Research Division, Korea Institute of Oriental Medicine, Daejeon, South Korea; ^2^KM Convergence Research Division, Korea Institute of Oriental Medicine, Daejeon, South Korea; ^3^Department of Korean Internal Medicine, College of Korean Medicine, Wonkwang University, Iksan, South Korea; ^4^Department of Rehabilitation Medicine and Institute of Wonkwang Medical Science, Wonkwang University School of Medicine, Iksan, South Korea

**Keywords:** acupuncture, TCM, evidence map, evidence synthesis, overview, stroke rehabilitation, systematic review

## Abstract

Evidence mapping of systematic reviews (SRs) systematically and comprehensively identifies, organizes, and summarizes the distribution of scientific evidence in a field. The aim of this evidence map is to provide a synopsis of the best clinical practices and interventions in stroke rehabilitative care and to identify areas with a paucity of evidence to guide future research. PubMed, EMBASE, CDSR, six Korean databases, and two Chinese databases were searched for SRs evaluating the effectiveness of any stroke rehabilitation intervention through October 2021. The quality of the SRs was assessed using AMSTAR 2. A bubble plot was used to graphically display clinical topics, the number of articles, the number of patients included, confidence, and effectiveness. In total, ninety-five SRs were identified; however, after methodological analysis, only 48 had sufficient quality to be included. In total, forty-eight SRs were included in the evidence mapping. The overall search identified SRs from 2015 to 2021. A total of four SRs focused on post-stroke cognitive impairment, whereas the other forty-four SRs focused on post-stroke motor function. In total, nineteen different traditional Chinese medicine (TCM) intervention modalities were included. Acupuncture was the most commonly used treatment. Overall, the quality of the included SRs was low or very low. Most SRs concluded that TCM interventions may have potential benefits in stroke rehabilitation. The results were more promising when acupuncture was used for shoulder–hand syndrome. However, the identified reviews cautioned that firm conclusions cannot be drawn. The evidence map provides a visual overview of the research volume and content involving TCM interventions in stroke rehabilitation. Evidence mapping can facilitate the process of knowledge translation from scientific findings to researchers and policymakers and possibly reduce waste in research.

## Introduction

Stroke is the second leading cause of death and long-term disability worldwide (1). Despite advances in modern medicine and medications, stroke remains a burden affecting disability-adjusted life years (2). Strokes can cause significant impairments that include different degrees of cognitive and behavioral dysfunction, paralysis, dysphagia, aphasia, and motor dysfunction (3). The mortality rate of stroke has been gradually decreasing, but the disability rate of stroke remains high (4). Appropriate stroke rehabilitation treatment is essential to minimize patient disability, promote the return to social activities, and improve quality of life. However, while modern medicine lacks effective treatment for this recovery period, traditional Chinese medicine (TCM) offers great possibilities (5).

TCM has been used for centuries in the treatment of stroke. Because of fewer side effects, TCM has often been sought to provide intervention therapies for the prevention of and rehabilitation from a stroke in China and Korea. Furthermore, TCM is popular not only in other parts of Asia but also in some Western countries, including USA and Australia. TCM mainly includes herbal medications, acupuncture, moxibustion, cupping, and tuina. More than 100 kinds of TCM interventions have been used to prevent and treat stroke (6). Acupuncture in particular is safe and improves cognitive function and depressive disorder in post-stroke patients (7). TCM has the merits of diminishing disability rates, boosting the quality of life, having low toxicity and side effects, and having low therapy costs for patients in post-stroke recovery (8). However, evidence for the efficacy and safety of these interventions remains inconsistent and uncertain. Meanwhile, the quality of the methodology and evidence in the field remains unknown.

There is a vast amount of scientific literature proposing treatment approaches for stroke rehabilitation. Systematic reviews (SRs) are one of the options used to organize and critically assess published studies and summarize the results of the evidence from healthcare-related primary studies to answer specific research questions. Evidence mapping of SRs systematically and comprehensively identifies, organizes, and summarizes the distribution of scientific evidence in a field, aiming to identify gaps in knowledge and future research needs. This evidence mapping aims to provide a synopsis of the best clinical practices and interventions in stroke rehabilitative care and identify areas with a paucity of evidence to guide future research.

## Methods

### Study Design

Evidence mapping is not associated with an official standardized method (9). The approach in this study was adopted from the methodology that Solloway et al. used in the “Evidence Map of Tai Chi” (10).

### Electronic Searches and Search Strategy

SRs were searched in 11 databases, including PubMed, EMBASE, and the Cochrane Database of Systematic Reviews (CDSR), and also six Korean databases [Korea Med, the Oriental Medicine Advanced Search Integrated System (OASIS), DBpia, the Korean Medical Database (KM base), the Research Information Service System (RISS) and the Korean Studies Information Services System (KISS)] and two Chinese databases [the China National Knowledge Infrastructure (CNKI) and Wang Fang], from database inception through October 2021. In addition, the reference lists of potentially eligible articles were searched manually to identify additional relevant articles.

The search terms used were based on the text words “systematic review” or “meta-analysis.” and “stroke rehabilitation,” and database-specific filters for SRs were used to develop the search strategy with no language restrictions (Supplementary Table 1).

### Inclusion Criteria

#### Design

Only SRs focusing on TCM interventions in stroke rehabilitation and summarizing primary research studies for all the clinical indications were included. In this study, we considered SRs containing at least one randomized controlled trial (RCT), which addressed the use of TCM in stroke rehabilitation.

#### Population

We examined trials, including adults (aged over 18 years) with a clinical diagnosis of stroke (all types, severity levels, and stages of stroke), paresis of the upper, lower, or both sets of limbs (motor function), and confirmed cognitive impairment as specified in each trial (cognitive impairments included disruptions in attention and concentration, memory, orientation, and/or executive functions).

#### Intervention and Comparators

All types of TCM interventions were considered, including but not limited to the following: acupuncture, electroacupuncture (EA), Chinese herbal medicine (CHM), moxibustion, tai chi, qigong, Chinese herbal bath, and tuina. Combination therapies incorporating TCM interventions were also included. Comparators included non-treatment, sham treatment, placebo treatment, and routine treatments (rehabilitation and positive interventions).

#### Outcomes

SRs reporting on patients' health outcomes were eligible for inclusion. SRs focused on provider outcomes, study design, or intervention features that did not report patient health outcomes were excluded.

### SR Selection

All scoping, rapid, critical, and narrative reviews were excluded. Two reviewers (T-YC and JJ) independently screened all the titles and abstracts and selected full-text articles to exclude irrelevant SRs. Disagreements were resolved through discussion and consensus, and an additional reviewer (MSL) was consulted. Where originals and updates of SRs by the same author group were available, only the most recent version was considered, and multiple publications of the same review were counted as one review, although data were extracted from all the available publications. If multiple reviews of similar clinical topics were identified, the most pertinent and best-performing SR was used for inclusion in evidence maps and was selected based on the results of the Assessing the Methodological Quality of Systematic Reviews 2 (AMSTAR 2) assessment.

### Data Extraction

All the articles were read by two independent reviewers (T-YC and JHJ), data were extracted from the articles based on predefined criteria, and a methodological quality assessment was conducted. Disagreements were resolved by consensus, and when necessary, an additional reviewer (MSL) participated in the discussion. Information on PICO (population, intervention, comparison, and outcomes), the number of RCTs included in each SR, summary effect estimates for main outcomes, overall risk of bias (ROB), publication bias, and conclusions (quoted from the original article) were extracted from the included SRs.

### Methodological Quality Assessment

The AMSTAR 2 tool was used to critically appraise the quality of reporting for each included SR. A validated 16-item instrument for critically appraising SRs that assesses the quality and bias using ratings of “yes,” “partial yes,” or “no” (11). Overall confidence in the results of an SR is rated according to the following four categories: “high” (no or one non-critical weakness), “moderate” (more than one non-critical weakness), “low” (one critical flaw with or without non-critical weaknesses), and “critically low” (more than one critical flaw with or without non-critical weaknesses).

### Evidence Mapping Presentation

We used the topics of the identified SRs to categorize the reviews. We presented the evidence mapping in tables describing the characteristics of the included SRs and a graphic display of the mapping based on bubble plots. Each bubble in the chart represents one included SR. The SR grouping into the clinical topics was drafted by one reviewer and discussed among the review team.

The chart displays information in four dimensions:

1) X-axis: stroke rehabilitative symptoms.

Stroke rehabilitative symptoms were classified. The studies were categorized into those assessing cognitive function and those assessing motor function.

2) Y-axis: AMSTAR 2 assessment/strength of findings/

Confidence was decided based on the results of the AMSTAR 2 assessment, and the reviews were classified into four categories as follows: “high,” “moderate,” “low,” or “critically low.”

3) Bubble size: number of primary studies included in the SR.

Each SR bubble size is proportional to the number of primary studies included in the SR evaluating the effects of a particular intervention.

4) Circle color: effect estimate.

The clinical effectiveness of the rating of the authors' conclusions and overall ROB are described in the selected SR. Clinical effectiveness was categorized as a green circle if “effective” (if effect estimates were significantly positive and the overall ROB was low), a blue circle if “potentially effective” (if effect estimates were significantly positive but the overall ROB was high), or a yellow circle if “unclear” (if effect estimates were negative or the overall ROB was unclear).

## Results

### Study Selection

**Figure 1:**
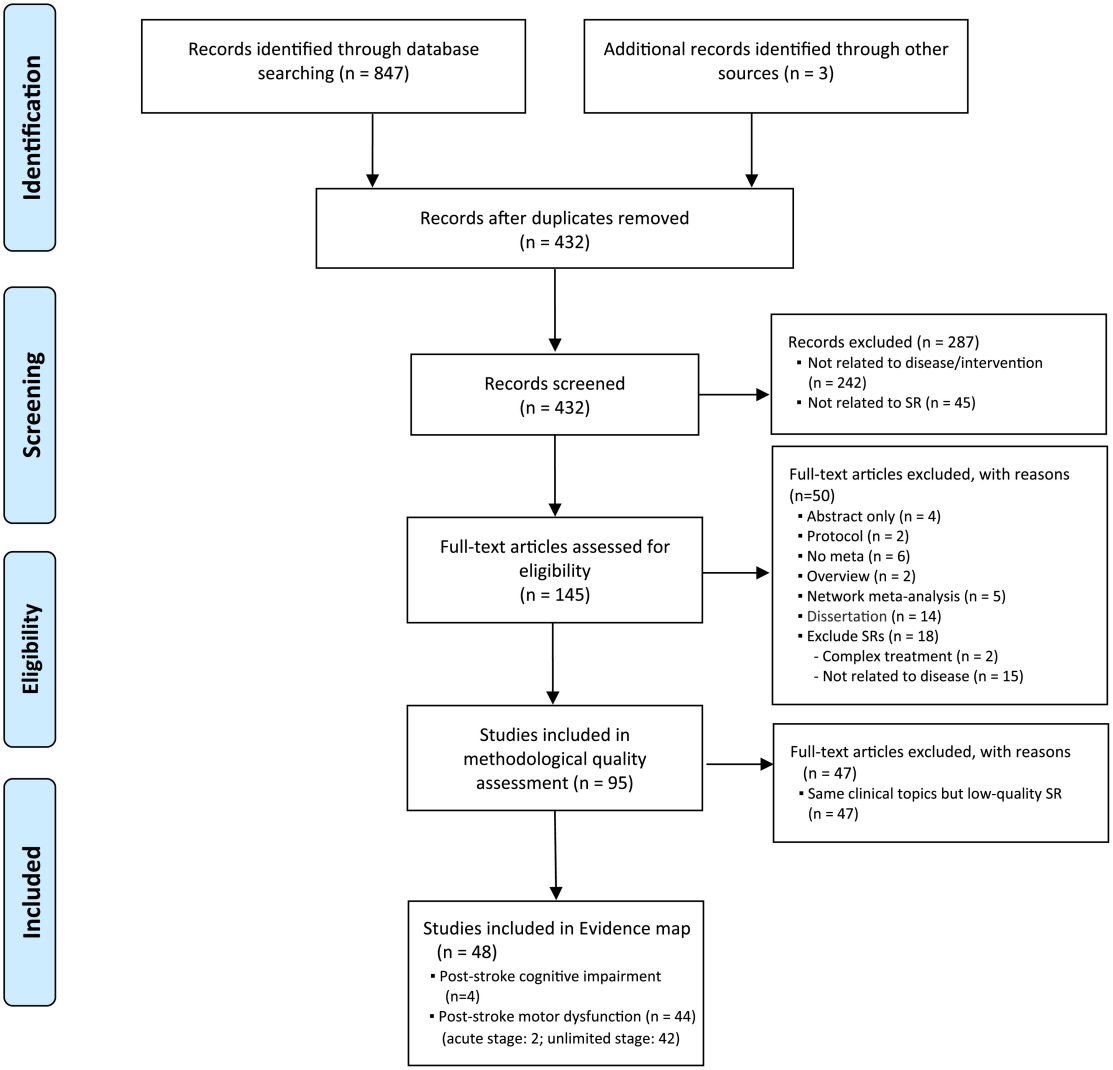
Study flow diagram. SR, systematic review.

The database search identified 847 potentially relevant studies. The research yielded 432 articles after removing duplicates. After title and abstract screening, 146 articles were obtained for the final full-text review. In total, ninety-five SRs were identified for potential inclusion; however, after methodological analysis, only 48 had sufficient quality to be included (Figure 1). Among the forty-eight studies that met the inclusion criteria for use in the evidence map, forty-six bubbles representing the unlimited stage and two bubbles representing the acute stage were created.

### Characteristics of the Included SRs

The forty-eight SRs (12–59) included a meta-analysis, and there were forty-five SRs from China and three SRs from Korea published between 2015 and 2021. There were thirty-six SRs published in Chinese, ten in English, and two in Korean. The main characteristics of the forty-eight SRs, including sample size, patient characteristics, interventions, and primary outcomes, are reported in Tables 1, 2. All the SRs contained only RCTs. The number of RCTs included in each SR ranged from 4 (18) to 41 (55). The number of patients included in each SR ranged from 310 (18) to 3,184 (20) adult individuals. The number of databases searched ranged from 2 to 12, and five SRs searched only the Chinese databases (27, 29, 31, 48, 59).

**Table 1:** Summary of the included systematic reviews of post-stroke cognitive impairment (PSCI).

**References, Country**	**Search date, No. of searched DB studies, No. of primary studies**	**Intervention**	**Comparator**	**Outcome**	**Effect estimates for main outcomes (meta-analysis)**	**Conclusion (quote from the original paper)**	**Overall risk of bias**	**Overall confidence**	**AMSTAR2 Rating overall confidence**
Zhou et al. (12), China	December 2019, 7, 37 (2,869)	AT + WM + CRT	WM + CRT	1) MMSE 2) MoCA	1) MD 2.88 [2.09, 3.66], *P* < 0.00001 2) MD 2.66 [1.95, 3.37], *P* < 0.00001	… effective in improving …	Cochrane ROB High	Potentially effective	Moderate
Zhan et al. (13), China	October 2016, 6, 14 (896)	EA + WM EA + CRT	WM CRT	1) MMSE 2) MoCA 3) P300 latency 4) P300 amplitude 5) FMA 6) ER 7) BI	1) MD 1.78 [0.24, 3.32], *P* = 0.02 2) MD 1.92 [0.96, 2.88], *P* <0.000 1 3) MD −11.01 [18.91, −3.11], *P* = 0.0006 4) MD 1.56 [1.14, 1.98], *P* < 0.00001 5) MD 10.74 [2.67, 18.82], *P* = 0.009 6) RR 1.37 [0.98, 1.91], *P* = 0.06 7) MD 6.38 [−2.41, 15.18], *P* = 0.15	… effective and safe for PSCI, which could improve cognitive function and motor function	Cochrane ROB High	Potentially effective	Low
Xiong et al. (14), China	May 2014, 6, 13 (1,113)	Scalp AT/EA Scalp AT/EA + WM Scalp AT/EA + CRT	WM CRT	1) MMSE 2) P300 latency	1) MD 2.22 [1.38, 3.07], *P* < 0.00001 2) MD −1.85 [−3.04, −0.66], *P* = 0.002	Insufficient…	Cochrane ROB High	Unclear	Critically low
Shen et al. (15), China	January 2018, 7, 16 (1,296)	HM HM + WM	WM	1) MoCA 2) MMSE 3) BI 4) NIHSS	1) HM + WM vs. WM: MD 2.57 [1.51, 3.63], *P* <0.0001 2) HM + WM vs. WM: MD 1.30 [0.45, 2.15], *P* = 0.003 (3 months); HM vs. WM: MD 1.58 [0.66, 2.51], *P* = 0.0008 (3 months) 3) HM + WM vs. WM: MD 12.36 [8.79, 15.92], *P* <0.0001 4) HM + WM vs. WM: MD −1.46 [−2.04, −0.86], *P* <0.0001	… potential advantages in improving …, and it also has certain efficacy in improving…	Cochrane ROB High	Potentially effective	Low

*ADL, activities of daily living; AT, acupuncture; BI, barthel index scale; CRT, cognitive rehabilitation; EA, electroacupuncture; ER, effective rate; FMA, fugl-meyer assessment; HM, herbal medicine; NCSE, neurobehavioral cognitive state examination total score; NIHSS, national institutes of health stroke scale; MD, mean difference; MESS, modified edinburgh stroke scale; MMSE, mini mental state examination; MoCA, montreal cognitive assessment scale; ROB, risk of bias; RR, risk ratio; RT, rehabilitation scale; SMD, standardized mean difference; WM, western medicine; WMD, weighted mean difference*.

**Table 2:** Summary of the included systematic reviews of post-stroke motor dysfunction.

**References, Country**	**Search date, No. of searched DB studies, No. of primary studies (total No. of patients)**	**Intervention**	**Comparator**	**Outcome**	**Effect estimates for main outcomes (meta-analysis)**	**Conclusion (quote from the original paper)**	**Overall risk of bias**	**Overall confidence**	**AMSTAR2 Rating overall confidence**
**Post-stroke shoulder pain**									
Lin et al. (16), China	February 2015, 7, 13 (869)	AT + RT	RT	1) VAS 2) FMA-U 3) ADL	1) MD 1.50 [0.92, 2.09], *P* < 0.00001 2) MD 6.38 [1.16, 11.60], *P* = 0.02 3) MD 11.25 [3.00, 19.49], *P* = 0.007	…more effective than….	Cochrane ROB High	Potentially effective	Critically low
Wei et al. (17), China	February 2019, 9, 18 (1,405)	Floating AT Floating AT + RT + UC	RT AT UC	1) SHSS 2) VAS 3) FMA	1) MD 1.75 [1.13, 2.38], *P* < 0.00001 2) MD 1.55 [1.24, 1.87], *P* < 0.00001 3) MD 4.24 [1.61, 6.67], *P* = 0.002	… effectively improve the upper limb function…	Cochrane ROB High	Potentially effective	Low
Lim and Lee (18), Korea	August 2014, 9, 4 (310)	BVA BVA+ Other treatment (AT, WM)	Saline injection AT UC	1) VAS 2) FMA	1) SMD 1.46 [0.30, 2.62], *P* = 0.01 2) SMD 1.60 [−0.53, 3.73], *P* = 0.14	… effective in relieving shoulder pain	Cochrane ROB High	Unclear	Critically low
Oh and Lee (19), Korea	September 2019, 7, 14 (861)	TN + Other treatment (RT, WM, EA, HM)	RT WM EA HM	1) VAS 2) ER	1) Chuna +HM vs. HM: MD −2.02 [−2.73, −1.32], *P* < 0.00001; Chuna + RT vs. RT: MD −1.49 [−1.95, −1.02], *P* < 0.00001 2) Chuna + RT vs. RT: RR 1.14 [1.04, 1.26], *P* = 0.007; Chuna +EA vs. EA: RR 1.07 [0.81, 1.42], *P* = 0.64	…statistically significant effect in pain reduction	Cochrane ROB High	Potentially effective	Low
**Post-stroke shoulder-hand syndrome**									
Liu et al. (20), China	January 2019, 9, 38 (3,184)	AT/EA + RT	RT	1) FMA 2) VAS	1) Overall: 8.01 [6.69, 9.33], *P* < 0.00001; EA + RT vs. RT: MD 9.08 [6.81, 11.35], *P* < 0.00001; AT + RT vs. RT: MD 7.80 [6.30, 9.30], *P* < 0.00001 2) Overall: MD −1.59 [−1.86, −1.32], *P* < 0.00001; EA + RT vs. RT: MD −1.50 [−1.84, −1.17], *P* < 0.00001; AT + RT vs. RT: MD −1.62 [−1.97, −1.28], *P* < 0.00001	… effective for motor function, pain relief and activities of daily living in stroke patients …	Cochrane ROB High	Effective	Low
Wang et al. (21), China	October 2018, 7, 23 (1,580)	EA+ RT	RT	1) ER 2) FMA 3) MBI 4) VAS	1) RR 1.12 [1.08, 1.17], *P* <0.0001 2) MD 7.36 [4.71, 10.02], *P* <0.0001 3) MD 16.09 [−7.52, 39.70], *P* <0.0001 4) MD −1.54 [−1.86, −1.21], *P* <0.0001	…has a significant effect…	Cochrane ROB High	Potentially effective	Low
Li et al. (22), China	March 2016, 7, 15 (981)	Jin's three-AT Jin's three-AT + Other treatment (RT, AT, Warm AT, WM)	RT AT WM	1) ER 2) FMA 3) VAS	1) J3N vs. Other treatment: RR 1.10 [0.98, 1.25], *P* = 0.11; J3N + Other treatment vs. Other treatment: RR 1.24 [1.12, 1.38], *P* <0.0001 2) MD −1.78 [−2.29, −1.28], *P* < 0.00001 3) MD 9.20 [8.50, 9.90], *P* < 0.00001	…effective to treat shoulder hand syndrome after stroke...	Cochrane ROB High	Potentially effective	Critically low
An (23) China	July 2016, 12, 6 (400)	Warm AT + Other treatment (AT, RT, UC)	AT + Other treatment (UC, RT)	1) VAS 2) FMA 3) STEF	1) MD −2.34 [−4.65, −0.02], *P* = 0.05 2) MD 6.87 [3.39, 10.35], *P* <0.01 3) MD −2.67 [−7.10, 1.76], *P* > 0.05	… can improve pain and edema & swelling, …. better than …	Cochrane ROB High	Unclear	Critically low
Wu et al. (24), China	January 2018, 8, 14 (1, 43)	Floating AT	EA RT	1) ER 2) FMA 3) VAS 4) SHSS	1) OR 5.42 [3.45, 8.52], *P* < 0.00001 2) MD 2.93 [−1.97, 7.83], *P* = 0.24 3) MD −1.46 [−1.87, −1.06], *P* = 0.00001 4) MD −0.45 [−0.67, −0.22], *P* = 0.0001	…better effect for the treatment of shoulder- hand syndrome after stroke…	Cochrane ROB High	Potentially effective	Low
Hou et al. (25), China	September 2019, 7, 10 (640)	Acupoint catgut embedding + UC	UC	1) ER 2) VAS 3) FMA 4) BI	1) OR 3.43, [1.97, 6.00], *P* <0.0001 2) MD −1.36 [−1.76, −0.96], *P* <0.0001 3) MD 8.19 [5.00, 11.39], *P* < 0.00001 4) MD 7.94 [4.53, 11.34], *P* < 0.00001	…could effective improve…	JBI High	Potentially effective	Low
Lin et al. (26), China	January 2017, 6, 15 (1,421)	Tuina + Other treatment (AT, RT, UC)	RT UC	1) FMA-U 2) VAS	1) MD 10.12 [9.62, 10.62], *P* < 0.00001 2) MD −1.68 [−1.91, −1.45], *P* < 0.00001	…can improve…	Cochrane ROB High	Potentially effective	Critically low
Zheng et al. (27) China	July 2018, 4 (only China), 12 (1,180)	Herbal socking + Other treatment (RT, AT, Tuina)	RT	1) ER 2) NRS 3) FMA 4) BI	1) RR 3.72 [2.61, 5.30], *P* < 0.00001 2) MD −1.55 [−1.80, −1.30], *P* < 0.00001 3) MD 7.85 [5.65, 9.51], *P* < 0.00001 4) MD 33.87 [16.67, 51.08], *P* <0.0001	…certain effect on….	Cochrane ROB High	Potentially effective	Low
Wang et al. (28), China	April 2019, 8, 12 (887)	Herbal fumigation + Other treatment (AT, RT)	RT AT RT + AT	1) ER 2) FMA 3) VAS	1) OR 3.11 [1.48, 6.52], *P* <0.003 2) MD 4.16 [2.90, 5.42], *P* < 0.00001 (2 wks); MD 6.01 [4.95, 7.08], *P* < 0.00001 (4 wks) 3) MD −0.76 [−0.82, −0.69], *P* < 0.00001 (Age > 60); MD −2.06, [−2.22, 1.90], *P* < 0.00001 (Age <60)	… can relieve limb pain, improve upper limb motor function and clinical efficacy	Cochrane ROB High	Potentially effective	Low
Guo et al. (29) China	December 2014, 4 (only China), 20 (1,399)	CHM CHM + Other treatment (AT, RT, WM, UC)	RT AT WM UC	1) ER 2) FMA 3) VAS	1) OR 2.55 [1.68, 3.85], *P* < 0.00001 2) MD 5.95 [3.88, 8.03], *P* < 0.00001 3) MD −2.43 [−3.51, −1.36], *P* < 0.00001	…improve the motor function of supper limb, reduce the pain of patients.	Jadad High	Potentially effective	Critically low
**Post-stroke hand spasm**									
Qi et al. (30), China	April 2018, 6, 10 (875)	AT AT + RT	RT	1) MAS 2) MBI 3) FMA 4) ADL	1) MD −0.97 [−1.16, 0.77], *P* <0.0001 2) MD 7.07 [3.96, 10.17], *P* < 0.00001 3) MD 2.55 [1.56, 3.54], *P* < 0.00001 4) MD 9.57 [4.63, 14.50], *P* = 0.0001	…certain therapeutic effect ….	Cochrane ROB High	Potentially effective	Low
**Post-stroke strephenopodia**									
Zhang et al. (31), China	February 2015, 5 (only China), 12 (787)	AT + RT	RT	1) ER 2) FMA 3) Ashworth 4) CSI	1) RR 1.19 [1.09, 1.31], *P* = 0.0001 2) MD 5.07 [4.18, 5.95], *P* < 0.00001 3) MD −0.68 [−0.91, −0.45], *P* < 0.00001 4) MD −0.58 [−1.18, −0.18], *P* = 0.007	…was effective	Cochrane ROB High	Potentially effective	Critically low
**Post-stroke motor dysfunction**									
Zhan et al. (32), China	November 2015, 5, 12 (889)	EA + Other treatment	RT Exercise AT	1) FMA-U 2) FMA-L 3) FMA 4) BI 5) NIHSS	1) MD −3.87 [−6.32, −1.42], *P* = 0.002 2) MD 2.80 [−3.29, −8.88], *P* = 0.37 3) MD 9.59 [8.93, 10.24], *P* < 0.00001 4) MD 8.91 [2.96, 14.86], *P* < 0.00001 5) MD 1.10 [0.94, 1.29], *P* = 0.22	…suggests that EA is effective and safe for recovery of patients with post stroke motor dysfunction	Cochrane ROB High	Potentially effective	Critically low
Lyu et al. (33), China	October 2017, 8, 21 (1,293)	Tai Chi + RT	RT	1) ADL 2) FMA 3) FMA-U 4) FMA-L 5) BBS 6) Holden scale 7) TUGT	1) MD 9.92 [6.82, 13.02], *P* < 0.00001 2) MD 4.49 [1.92, 7.06], *P* = 0.0006 3) MD 8.27 [4.69, 11.84], *P* <0.0001 4) MD 2.75 [0.95, 4.56], *P* = 0.003 5) MD 5.23 [3.42, 7.05], *P* < 0.00001 6) MD 0.61 [0.38, 0.85], *P* < 0.00001 7) MD 2.59 [1.76, 3.43], *P* < 0.00001	… beneficial effect on ADL, balance, limb motor function, and walking ability among stroke survivors	Cochrane ROB High	Potentially effective	Low
Zou et al. (34), China	December 2017, 8, 8 (822)	Baduanjin + Other treatment (RT, Balance training, Educational lessons)	RT Balance training Educational lessons	BBS	MD 2.39 [2.14, 2.65], *P* <0.001	… as an adjunctive and safe method may be conducive … achieve the best possible short-term outcome…	PEDro High	Potentially effective	Moderate
Zheng et al. (35), China	August 2014, 9, 16 (1,280)	AT	Usual care	1) ER 2) FMA 3) BI 4) NIHSS	1) OR 3.20 [2.01, 5.10], *P* <0.01 2) WMD 9.86 [6.34, 13.37], *P* < 0.00001 3) WMD 7.02 [3.17, 10.88], *P* = 0.0004 4) WMD −1.48 [−2.09, −0.88], *P* < 0.00001	…was effective	Cochrane ROB High	Potentially effective	Low
Zhan et al. (36), China	December 2016, 6, 19 (1,434) Acute stroke survivors within 14 days	EA + RT/WM	RT/WM	1) FMA 2) FMA-L 3) ER 4) ADL	1) EA vs. non-EA: WMD 10.79 [6.39, 15.20], *P* <0.001; EA plus RT plus WM vs. RT plus WM: MD 8.03 [5.17, 10.90], *P* <0.001 2) EA vs. non-EA: WMD 5.16 [3.78, 6.54], *P* <0.001 3) EA vs. non-EA: RR 1.13 [1.00, 1.27], *P* = 0.050 4) EA vs. non-EA: MD 1.37 [0.79, 1.96], *P* <0.001; EA plus RT plus WM vs. RT plus WM: 1.29 [0.55, 2.02], *P* <0.001	...provides new evidence for the effectiveness and safety...	Cochrane ROB High	Potentially effective	Moderate
**Post-stroke spasticity**									
Ye et al. (37), China	December 2015, 6, 30 (2,453)	AT + RT	RT	1) ER 2) MAS 3) CSI	1) OR 2.69 [2.08, 3.47], *P* <0.001 2) OR 2.47 [2.02, 3.02], *P* <0.001 3) MD −1.01 [−1.47, −0.54], *P* <0.001	… could be effective in decreasing spasticity after stroke, …	Cochrane ROB High	Potentially effective	Critically low
Qiu et al. (38), China	August 2020, 8, 16 (1,118)	Fire AT	AT	1) ER 2) Recover rate 3) FMA 4) MAS 5) BI 6) NDS	1) RR 1.51 [1.36, 1.66], *P* < 0.00001 2) RR 2.59 [1.75, 3.84], *P* < 0.00001 3) SMD 2.27 [1.40, 3.13], *P* < 0.00001 4) SMD 0.47 [0.18, 0.77], *P* = 0.002 5) SMD 1.46 [1.03, 1.90], *P* < 0.00001 6) SMD 0.90 [0.44, 1.35], *P* = 0.0001	… provide a better clinical effect than conventional AT, …	Cochrane ROB High	Potentially effective	Moderate
Yang et al. (39), China	February 2016, 10, 12 (878)	Warm AT Warm AT + RT	AT/EA AT/EA + RT	1) FMA 2) BI 3) ADL	1) EA: MD −0.53 [−0.75, −0.31], *P* < 0.00001; AT: SMD −0.66 [−1.19, −0.13], *P* = 0.01 2) EA: MD 7.70 [4.78, 10.63], *P* < 0.00001; AT: MD 7.78 [4.36, 11.21], *P* < 0.00001 3) EA: MD 12.64 [11.71, 13.57], *P* < 0.00001; AT: MD 3.79 [−3.06, 10.65], *P* = 0.28	… promising intervention to reduce limb spasm as well as improve motor function and daily living activities …	Cochrane ROB High	Potentially effective	Critically low
Cai et al. (40), China	February 2018, 12, 35 (2,457)	HM	RT	1) MAS-U 2) MAS-L 3) FMA 4) FMA-U 5) FMA-L 6) BI	1) Oral HM: SMD −1.79 [−3.00, −0.57], *P* = 0.004; Topical HM: SMD −1.06 [−1.40, −0.72], *P* < 0.00001 2) Oral HM: SMD −1.01 [−1.43, −0.59], *P* < 0.00001; Topical HM: SMD −1.16 [−1.83, −0.49], *P* = 0.0007 3) Oral HM: SMD 12.14 [1.57, 22.71], *P* = 0.02; Topical HM: MD 5.56 [2.38, 8.74], *P* = 0.0006 4) Oral HM: SMD 7.64 [−1.29, 16.57], *P* = 0.09; Topical HM: MD 5.88 [4.09, 7.68], *P* < 0.00001 5) Oral HM: SMD 4.03 [1.90, 16.57], *P* = 0.0002 6) Oral HM: MD 13.15 [4.37, 21.93], *P* = 0.003; Topical HM: MD 12.01 [2.81,21.22], *P* = 0.01	… suggests that HM appears to be a well-tolerated therapy for patients with PSS	High	Potentially effective	Low
Yan et al. (41), China	April 2014, 8, 20 (1,720)	Tuina Tuina + Other treatment (AT, WM, RT, BT)	UC WM RT Tuina	1) MAS 2) FMA 3) MBI	1) MD −1.42 [−3.19, −0.36], n.r. 2) MD 4.72 [2.96, 6.48], n.r. 3) MD 10.39 [8.92, 11.86], n.r.	… improving motor function and daily life has significant curative effect, …	Cochrane ROB High	Potentially effective	Critically low
**Post-stroke spastic hemiplegia**									
Fan et al. (42), China	July 2019, 9, 38 (2,628)	AT/EA AT/EA + RT	RT	1) FMA 2) ASS 3) BI	1) MD 8.43 [6.57, 10.28], *P* < 0.00001 2) MD −0.46 [−0.65, −0.27], *P* < 0.00001 3) MD 8.32 [5.30, 11.35], *P* < 0.00001	… be a safe and effective adjuvant therapy …	Cochrane ROB High	Potentially effective	Moderate
Li and Wang (43), China	March 2020, 7, 11 (879)	EA+ RT	RT AT	1) ER 2) FMA 3) MAS 4) MBI 5) NDS 6) CSI	1) RR 1.27 [1.16, 1.40], *P* < 0.00001 2) MD 9.09 [7.47, 10.71], *P* < 0.00001 3) MD −0.36 [−0.57, −0.16], *P* = 0.0005 4) MD 6.85 [5.16, 8.53], *P* < 0.00001 5) MD −2.61[−3.01, −2.20], *P* < 0.00001 6) MD −1.11 [−1.60, −0.62], *P* < 0.00001	… certain effect on spastic paralysis after stroke	Cochrane ROB High	Effective	Low
You et al. (44), China	February 2018, 6, 14 (918)	Fire AT Fire AT + Other treatment (UC, RT)	AT/EA AT/EA + Other treatment (UC, RT, moxa)	1) ER 2) FMA 3) BI 4) MAS 5) CSI 6) NDS	1) OR 2.87 [1.94, 4.25], *P* < 0.00001 2) SMD 1.00 [0.51, 1.50], *P* <0.0001 3) SMD 1.42 [0.71, 2.12], *P* <0.0001 4) SMD −0.67 [−1.06, −0.27], *P* = 0010 5) SMD −0.99 [−1.94, −0.03], *P* = 0.04 6) SMD −0.83 [−1.49, −0.17], *P* = 0.01	… effective and safe treatment for spastic paralysis after stroke	Cochrane ROB High	Potentially effective	Low
Xie et al. (45), China	November 2019, 4, 8 (690)	Needle-Knife Needle-knife + Other treatment (RT, HM)	RT RT + Other treatment (WM, AT)	1) ER 2) FMA 3) CSI	1) RR 1.20 [1.10, 1.32], *P* <0.0001 2) MD 7.95 [4.85, 11.05], *P* < 0.00001 3) MD −1.79 [−2.67, −0.92], *P* <0.0001	… can improve spasticity and improve clinical efficacy	Cochrane ROB High	Potentially effective	Low
Yu and Wu (46), China	February 2018, 6, 13 (1148)	Warm AT	EA AT	1) ER 2) BI 3) FMA 4) MAS 5) MAS-U 6) MAS-L	1) RR 1.25 [1.15, 1.37], *P* < 0.00001 2) MD 12.75 [12.14, 13.36], *P* < 0.00001 3) MD 9.42 [8.63, 10.21], *P* < 0.00001 4) MD −0.57[−0.74, −0.40], *P* < 0.00001 5) MD −0.47 [−0.66, −0.28], *P* < 0.00001 6) MD −3.21 [−3.51, −2.91], *P* < 0.00001	… can improve the curative effect of spastic hemiplegia patients after stroke and accelerate the RT	Cochrane ROB High	Potentially effective	Critically low
Wen et al. (47), China	May 2018, 7, 8 (689)	Jin's three-AT Jin's three-AT + RT	RT	1) NDS 2) FMA 3) ADL 4) CSI 5) FCA	1) MD −3.36 [−3.93, −2.79], *P* < 0.00001 2) MD 15.09 [12.19, 18], *P* < 0.00001 3) MD 13.61 [10.72, 16.51], *P* <0.0001 4) MD −1.44 [−2.05, −0.83], *P* < 0.00001 5) MD 10.22 [6.94, 13.50], *P* < 0.00001	… can improve the treatment efficiency …	Cochrane ROB High	Potentially effective	Low
Chen and Tan (48) China	November 2015, 3 (only China), 10 (732)	CHM (Shaoyao gancao decoction) CHM (Shaoyao gancao decoction) + Other treatment (RT, WM, herbal umigation)	RT WM	1) FMA 2) ER 3) BI	1) MD 9.22 [6.31, 12.14], *P* < 0.00001 2) MD 7.11 [4.34, 9.89], *P* < 0.00001 3) RR 1.15 [1.05, 1.27], *P* = 0.003	… has a certain curative effect on the treatment of …	Cochrane ROB High	Potentially effective	Critically low
Ma et al. (49), China	June 2015, 8, 11 (765)	Moxa Moxa + Other treatment (AT, UC, RT)	AT RT AT + RT	1) MAS 2) FMA 3) BI 4) ER	1) SMD −2.06 [−3.58, –.54], *P* = 0.008 2) MD 11.29 [7.23, 15.36], *P* < 0.00001 3) SMD 1.05 [0.25, 1.86], *P* = 0.010 4) RR 1.23 [1.07, 1.41], *P* = 0.004	… can relieve spasm and improve the motion ability …	Cochrane ROB High	Potentially effective	Critically low
Fan et al. (50), China	July 2014, 7, 16 (1,098)	Tuina Tuina + Other treatment (Bobath therapy, UC, Moxa, RT)	AT RT	1) FMA 2) MAS 3) BI 4) NDS 5) CSI	1) MD 4.59 [1.81, 7.36], *P* = 0.001 2) MD −0.37 [−0.64, −0.10], *P* = 0.006 3) MD 6.15 [−1.33, 13.62], *P* = 0.11 4) MD −2.33 [−7.24, 2.58], *P* = 0.35 5) MD 0.62[−1.10, −0.14], *P* = 0.01	…can improve the conditions of post stroke spastic hemiplegia	Cochrane ROB High	Potentially effective	Critically low
**Post-stroke hemiplegia**									
Chen et al. (51), China	December 2018, 5, 9 (762)	AT + Other treatment (Tuina, HM, Cupping, WM, Moxa, venesection)	WM HM	ER	OR 3.71 [2.09, 6.58], *P* < 0.00001	… a positive effect on the improvement of ER …	Cochrane ROB High	Potentially effective	Moderate
Liu and Wang (52) China	December 2015, 6 (385)	Eyes AT	WM	ER	OR 5.05 [3.13, 8.18], *P* < 0.00001	… more effective than …	Cochrane ROB High	Unclear	Critically low
Zhang et al. (53), China	March 2019, 6, 12 (1,259)	Herbal fumigation + Other treatment (WM, RT)	WM RT	1) ER 2) FMA 3) BI 4) MBI	1) RR 1.26 [1.17, 1.34], *P* < 0.00001 2) MD 9.24 [6.27, 12.22], *P* < 0.00001 3) MD 9.38 [6.45, 12.30]], *P* < 0.00001 4) MD 9.27 [5.80, 12.73], *P* < 0.00001	… can improve the clinical effect …, and is better than WM alone or RT	Cochrane ROB High	Potentially effective	Low
Gou et al. (54), China	January 2018, 8, 8 (765)	CHM (Buyang huanwu decoction) + AT/EA	HM (Buyang huanwu decoction)	1) ER 2) BI	1) OR 3.89 [2.58, 5.86], *P* < 0.00001 2) SMD 2.35 [2.04, 2.66], *P* < 0.00001	… positive effect on the improvement of activity of daily life, …	Jadad High	Effective	Low
Ji and Guan (55), China	March 2019, 8, 41 (3,145)	Moxa + Other treatment (RT, AT, WM, UC)	WM AT RT UC	1) ER 2) NIHSS 3) FMA 4) BI	1) RR 1.26 [1.14, 1.39], *P* < 0.00001 2) MD −2.19 [−2.62, −1.76], *P* < 0.00001 3) MD 16.03 [11.12, 20.94], *P* = 0.33 4) SMD 1.11 [0.92, 1.30], *P* = 0.72	… has a positive effect on the improvement of motor function, … some of the results are heterogeneous	Jadad High	Potentially effective	Moderate
Lee et al. (56), Korea	October 2019, 11, 11 (863)	Daoyin Daoyin + RT	RT Brunnstrom's movement	1) FMA 2) MBI 3) NIHSS	1) Daoyin vs. RT: SMD 2.80[2.31, 3.30], *P* < 0.00001; Daoyin + RT vs. RT: SMD 0.81[0.11, 1.51], *P* = 0.02 2) Daoyin vs. RT: SMD 1.87 [0.57, 3.17], *P* = 0.005; Daoyin + RT vs. RT: SMD 0.89[0.61, 1.17], *P* <0.0001 3) Daoyin + RT vs. RT: SMD −0.96 [−1.22, −0.07], *P* <0.0001	…effects in functional recovery and in enhancing the independence of daily living activities for stroke patients	Cochrane ROB High	Potentially effective	Low
Wang et al. (57), China	n.r., 8, 8 (408)	Tai Chi Tai Chi + RT	RT	1) BBS 2) FMA 3) FAC	1) SMD 2.49 [0.90, 4.07], *P* = 0.002 2) SMD 1.71 [1.21, 2.20], *P* = 0.00001 3) SMD 0.20 [−1.20, 0.81], *P* = 0.70	… positive effect on the improvement of …	Jadad High	Potentially effective	Low
Lin and Liu (58), China	October 2014, 9, 6 (410) Acute stage	AT/EA	RT UC	MA	MD 6.71 [5.51, 7.90], *P* < 0.00001	… positive effect on the improvement of FMA, …	Cochrane ROB High	Potentially effective	Moderate
**Post-stroke thalamic pain**									
Bu and Ren (59), China	April 2016, 3 (only China), 10 (627)	AT/HM AT/HM + WM	WM	1) ER 2) Degradation rate	1) OR 3.32 [2.25, 4.90], *P* < 0.00001 2) OR 0.24 [0.08, 0.69], *P* = 0.009	… effective for post stroke thalamic pain …	Jadad High	Potentially effective	Critically low

*ACE, Acupoint catgut embedding; ADL, Activities of Daily Living; AT, acupuncture; ASS, Ashworth scale for spasticity; BBS, Berg Balance Scale; BDJ, Baduanjin; BI, Barthel Index scale; BMIT, Boston Motor Inventory Test; BT, Bobath therapy; BVA, Bee venom acupuncture; CHM, Chinese Herbal medicine; CSI, clinical spasm index; DB, databases; DGI, Dynamic Gait Index; DN, dry needing; DY, Daoyin; EA, Electroacupuncture; EAT, Eyes acupuncture; ER, Effective Rate; FAC, functional ambulation category scale; FAT, Floating acupuncture; FCA, functional comprehensive assessment; FIM, Functional Independence Measure; FNA, Fire-needle acupuncture; FMA, Fugl-Meyer Assessment; FMA-L, Fugl-Meyer Assessment of Lower Limb; FMA-U, Fugl-Meyer Assessment of Upper Limb; HF, Herbal fumigation, HM, Herbal medicine; HS, Herbal socking; JBI, Joanna Briggs Institute's critical appraisal tool; JTA, Jin's three-needle acupuncture; NDS, neurological functional defect scores; NKA, Needle knife acupuncture; NI, neurological impairment; NIHSS, National Institutes of Health Stroke Scale; NR, Not reported; NRS, numerical rating scale; MAS, The modified ashworth scale; MAS-L, The modified ashworth scale of Lower Limb; MAS-U, The modified ashworth scale of Upper Limb; MBI, Modified Barthel Index scale; MESS, modified Edinburgh Stroke Scale; MD, mean difference; MMSE, Mini Mental State Examination; MoCA, Montreal Cognitive Assessment Scale; Moxa, Moxibustion; OR, odds ratio; PEDro, Physiotherapy Evidence Database scale; PSS, post-stroke spasticity; ROB, risk of bias; RR, risk ratio; RT, rehabilitation scale; SAT, Scalp acupuncture; SF-36, 36-Item Short Form Health Survey; SHSS, shoulder-hand syndrome scale; SS-QOL, Stroke Specific Quality of Life Scale; SMD, standardized mean difference; SSS, Scandinavian Stroke Scale; SPBB, Short Physical performance Battery for Balance; STEF, simple test for evaluating hand function; TC, Taichi; TCM, Traditional Chinese Medicine; TCE, traditional Chinese exercises; TUGT, timed-up-and-go test; UC, usual care; QoL, Quality of Life; VAS, Visual analog scale; WM, western medicine*.

### Stroke Rehabilitative Symptoms Included

This article is based on multiple SRs. Four SRs focused on post-stroke cognitive impairment (Table 1), whereas the other 44 SRs focused on post-stroke motor function (Table 2), with shoulder–hand syndrome (*n* = 10) being the most frequent among them, followed by shoulder pain (*n* = 4), hand spasm (*n* = 1), strephenopodia (*n* = 1), motor dysfunction (*n* = 5), spasticity (*n* = 5), spastic hemiplegia (*n* = 9), hemiplegia (*n* = 8), and thalamic pain (*n* = 1).

### Intervention Component Description

In total, nineteen different TCM intervention modalities were included: acupuncture (*n* = 11), EA (*n* = 5), eye acupuncture (*n* = 1), floating acupuncture (*n* = 2), fire-needle acupuncture (*n* = 2), Jin's three-needle acupuncture (*n* = 2), scalp acupuncture (*n* = 1), warm-needle acupuncture (*n* = 3), appoint catgut embedding (*n* = 1), bee venom acupuncture (*n* = 1), needle knife acupuncture (*n* = 1), moxibustion (*n* = 2), CHM (*n* = 5), herbal fumigation (*n* = 2), herbal socking (*n* = 1), taichi (*n* = 2), baduanjin (*n* = 1), daoyin (*n* = 1), and tuina (*n* = 4). Descriptions of the TCM interventions are described in Supplementary Table 2.

### Quality of Included Systematic Reviews

Most SRs used the Cochrane handbook for risk or quality assessment. ROB was assessed by the Jadad scale in five SRs (29, 54, 55, 57, 59), while two SRs used the Joanna Briggs Institute's (JBI) critical appraisal tool (25), and two SRs used the Physiotherapy Evidence Database (PEDro) scale (34). Regarding quality assessment for evaluating the overall confidence level of each review, most studies showed moderate to critically low quality (Supplementary Table 3, Supplementary Figure 1). The lowest scores were on item 7 (none of the studies provided a list of excluded studies and justified the exclusions), item 2 (none of the studies reported justifications for any significant deviations from the protocol), and item 16 (5 studies did not report any potential sources of conflicts of interest). Overall confidence was rated as “moderate” for 8 SRs, “low” for 24 SRs, and “critically low” for 16 SRs.

### Effectiveness

Overall confidence was considered with respect to overlapping diseases. The conclusions were reflected in individual SRs and confirmed through an internal review. We evaluated the effectiveness, literature size, and confidence level for each intervention identified in the SRs.

#### Effective

The effects of TCM interventions in stroke rehabilitation, indicated by statistically significant pooled treatment effects in an SR (*n* = 1) and based on a substantial number of research studies, included findings with acupuncture for shoulder–hand syndrome (20).

#### Potentially Promising Effects

Promising effects of TCM interventions in stroke rehabilitation, indicated by statistically significant pooled treatment effects in the SRs (*n* = 43) and based on a substantial number of research studies, included acupuncture for cognitive impairment (12), EA for motor dysfunction (36), CHM for spasticity (40), and moxibustion for hemiplegia (55). Most SRs reached the conclusion that there may be potential benefits of TCM interventions in stroke rehabilitation.

#### Unclear Effect

The map includes a small number of SRs (*n* = 4) that provided evidence of the potential lack of effectiveness of TCM interventions in stroke rehabilitation for clinical indications across more than one included study: scalp acupuncture for cognitive impairment (14), bee venom acupuncture for shoulder pain (18), warm-needle acupuncture for shoulder–hand syndrome (23), and eye-acupuncture for hemiplegia (52). These promising results are, however, compromised by the low-quality overall of the clinical trials. The identified reviews cautioned that firm conclusions cannot be drawn.

### Evidence Map

**Figure 2:**
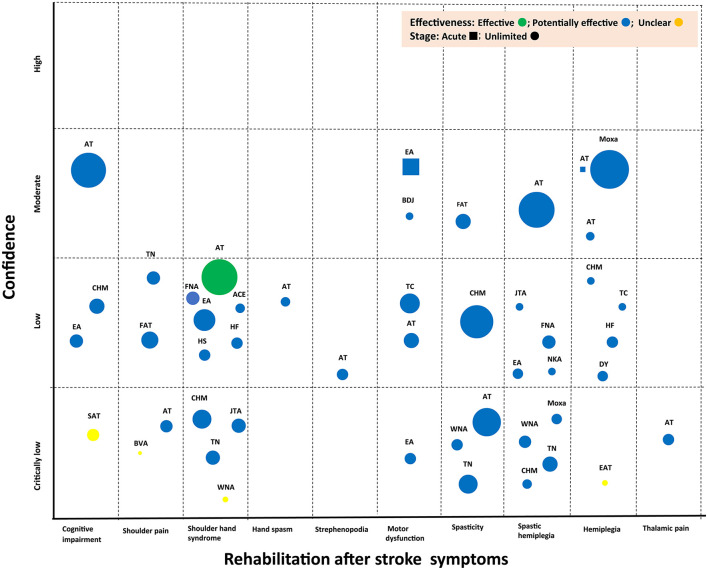
Evidence map of TCM for stroke rehabilitation. ACE, acupoint catgut embedding; AT, acupuncture; BDJ, Baduanjin; BVA, bee venom acupuncture; CHM, Chinese herbal medicine; DY, daoyin; EA: electroacupuncture; EAT, eyes acupuncture; FAT, floating acupuncture; FNA, fire-needle acupuncture; HF, herbal fumigation; HS, herbal socking; JTA, Jin's three-needle acupuncture; NKA, needle knife acupuncture; SAT, scalp acupuncture; TC, Taichi; TN, tuina; WNA, warm-needle acupuncture.

Figure 2 presents the results of the evidence mapping process. The evidence map displays each of the 48 included SRs as 48 bubbles. As noted in the Materials and Methods Section, the bubble label represents the TCM intervention in that review. The bubble size represents the effect of the number of included primary studies in the context of stroke rehabilitation. Primary studies may have been included in multiple SRs. Each bubble was plotted based on the effect of the TCM intervention in stroke rehabilitation (color) in the space defined by the symptoms of stroke rehabilitation (x-axis) and the strength of the findings for the TCM intervention in stroke rehabilitation (y-axis). The evidence tables provide details of the included SRs (Tables 1, 2).

The SR authors concluded across the identified studies that TCM interventions in stroke rehabilitation improved outcomes of interest; however, the number of existing studies in the identified topic areas was small in all of the identified topic areas. The evidence mapping showed that only a limited number of TCM interventions have been assessed in stroke rehabilitation and that the clinical evidence for these interventions is inconclusive, indicating a need for more original research in this area (Figure 2).

## Discussion

The 48 published SRs included in our evidence map provide a comprehensive overview of the evidence for TCM interventions in stroke rehabilitation published between 2015 and 2021. The evidence compiled by this overview indicated that TCM interventions (acupuncture, EA, CHM, moxibustion, etc.), in combination with conventional interventions, could improve cognitive and motor function. The results of this evidence map showed that in-line with available evidence, there is a sparsity of SRs evaluating TCM interventions in the context of stroke rehabilitation (cognitive and motor function). Furthermore, acupuncture can be more effective and safer than rehabilitation training in the treatment of post-stroke shoulder–hand syndrome (20). Some identified SRs included a large number of RCTs, but they addressed very broad topics, such as post-stroke motor dysfunction (32–36). On the other hand, evidence on the role of bee venom acupuncture in a number of specific conditions (post-stroke shoulder pain) is very limited due to the small number of published studies (18). The main beneficial treatment reported by the authors for patients with unlimited stage stroke was acupuncture with shoulder–hand syndrome. Evidence about the benefits of treatments for acute-stage stroke rehabilitation is lacking.

This evidence map describes the research foci that were reported in the existing SRs and displays the gaps in evidence so that areas that should be prioritized in future research can be identified. However, this evidence map is unable to answer more refined questions, such as what the best TCM interventions for specific applications and the differences between health services are. To advance our evidence-based knowledge of TCM, we should collect more data on the effectiveness of TCM for rehabilitating stroke symptoms and patient populations through meta-analyses across primary studies. In addition, the large number of treatments that were classified as having potential effectiveness warrants additional primary studies. More studies have been published in some of the areas of interest included in the unclear evidence category, and the currently available SRs must be updated. As there might be other efficient ways of drawing evidence maps, further research should also include developing evidence maps of other research designs.

The evidence map has several limitations. First, most publications are from mainland China and are written in Chinese, which eliminated their inclusion in this mapping of available evidence published in any other language. The generalization of these results to other countries might be limited. Second, the analysis was based on published SRs, and primary studies contributed to more than one included SR. Furthermore, individual review conclusions may have been limited by the quality of the primary studies and susceptible to publication and outcome reporting bias. There may be clinical trials that were included in more than one SR that might have an impact on the synthesized findings. Third, we attempted to retrieve possible eligible studies through comprehensive searches of numerous databases regardless of publication language, but there may have been missed trials related to this topic. Finally, the methodological quality of most included SRs scored “low” and “critically low.” More high-quality RCTs and SRs are needed to support clinical decision-making about the use of TCM interventions in stroke rehabilitation regimens. These findings highlight the need to conduct future research focusing on new treatments and addressing knowledge gaps in this field, and increased efforts are required to improve the methodological quality and reporting process of SRs on treatments to be used in stroke rehabilitation.

Stroke prevention and treatment remain a challenge worldwide. In China and Korea, many stroke patients are treated using traditional medicine, and there have been reports on their functional recovery (60, 61). Most alternative therapies are of unproven benefit in rehabilitation. Well-conducted trials are needed to better define the role of alternative therapies in the process of post-stroke recovery. In addition to future studies, better health education and rehabilitation services are also required.

## Conclusion

This evidence map summarized, organized, and provided a visual overview of the currently available research volume and content related to TCM interventions during stroke rehabilitation involving cognitive function and motor function. This visualization facilitates an easy and engaging overview and suggests evidence mapping as a useful tool for a large array of stakeholders and for informing policy and clinical decision-makers. Our results provide policy and clinical decision-makers guidance regarding the interpretation of the current state of evidence regarding the effectiveness of TCM interventions in stroke rehabilitation.

## Data Availability Statement

The original contributions presented in the study are included in the article/Supplementary Material, further inquiries can be directed to the corresponding author.

## Author Contributions

T-YC and MSL conceptualized the study and wrote the original draft. T-YC, JHJ, and MSL contributed to methodology. T-YC and J-MY contributed to the software and resources. HWL and MCJ validated the study and investigated the study. T-YC and JHJ contributed to formal analysis. JHJ and HWL contributed to data curation. JHJ, HWL, J-MY, and MCJ contributed to writing, reviewing, and editing the manuscript. T-YC visualized the study. MCJ and MSL contributed to supervision and funding acquisition. HWL contributed to project administration. All the authors read and approved the final manuscript.

## Funding

This study was supported by the Korea Health Industry Promotion Agency's Health and Medical Technology R&D Project (Grant No. HI20C1951) and the Korea Institute of Oriental Medicine (KSN2021210 and NHT2012480). The funders had no role in the study design, data collection and analysis, decision to publish, or preparation of the manuscript.

## Conflict of Interest

The authors declare that the research was conducted in the absence of any commercial or financial relationships that could be construed as a potential conflict of interest.

## Publisher's Note

All claims expressed in this article are solely those of the authors and do not necessarily represent those of their affiliated organizations, or those of the publisher, the editors and the reviewers. Any product that may be evaluated in this article, or claim that may be made by its manufacturer, is not guaranteed or endorsed by the publisher.
