# Supplementary material for: Traditional Chinese Medicine Interventions in the Rehabilitation of Cognitive and Motor Function in Patients With Stroke: An Overview and Evidence Map
Source: Front Neurol. 2022 May 17;13:885095. doi: 10.3389/fneur.2022.885095 (PMC9152210; doi:10.3389/fneur.2022.885095)
Supplement: Supplementary file 1 [file Data_Sheet_1.PDF]

Supplementary Table 1. Search strategy

**PubMed**	
1	stroke OR cerebrovascular accident OR hemiplegia OR hemiparesis OR hemiparetic
2	rehabilitation
3	cognitive impairment
4	motor OR movement OR motion OR mobility OR function
5	1 AND (2 OR 3 OR 4)
6	TCM OR Traditional Chinese Medicine OR Acupuncture OR Electroacupuncture OR Chinese herbal medicine OR moxibustion OR taichi OR qigong OR tuina
7	meta-analysis AND systematic review
8	5 AND 6 AND 7
**CNKI**	
1	脑卒中 OR 中风
2.	康复 OR 后 OR 认知障碍 OR 运动功能障碍
3.	1 AND 2
4.	中医 OR 中药 OR 针灸 OR 电针 OR 中草药 OR 艾灸 OR 太极 OR 气功 OR 推拿
5.	系统评价 OR Meta分析 OR 荟萃分析
6.	1 AND 4 AND 5
**Korea Med**	
	(중풍 AND (재활 OR 인지 OR 운동)) AND (체계적 문헌고찰 OR 메타분석

Supplementary Table 2. Definitions of different TCM intervention therapies.

**Type of TCM intervention**	**Abbreviation**	**Definitions**
Acupuncture	AT	Needle insertion into acupuncture points. Needling promotes Qi in the meridians.
Electroacupuncture	EA	Traditional acupuncture connected with electric stimulation apparatus, which is conducted by inserting acupuncture needles into the acupoints and then changing electric stimulation parameters, including the stimulation frequency, current intensity, pulse width, and pulse interval.
Eyes acupuncture	EAT	An involves fine-needle acupuncture, and may also include embedding catgut at acupoints and acupressure applied around the orbit of the eye.
Floating acupuncture	FAT	Also called Fu's Subcutaneous Needle (FSN), is an innovation for the treatment of myofascial pain and trigger points based on the research.<br>Originating from Traditional Chinese Medicine, FSN does not follow the rules and principles of TCM and the chosen insertion points do not coincide with traditional acupuncture points. The similarities are limited to the distal insertion of the needle to the affected area, the needle itself being a non-injection needle, and the fact that both needles are manipulated and act on soft connective tissue. FSN abstains from the muscle and deep fascia layers and is confined to only the subcutaneous layer where collagen fiber are most abundant.
Fire-needle acupuncture	FNA	An acupuncture technique that involves quickly inserting a red hot needle into acupuncture points on the body. Fire needling combines conventional acupuncture and cauterization with heated needles.
Jin's three-needle acupuncture	JTA	JTN is a kind of acupuncture therapy characterized by its special way of choosing treatment points. An acupuncture prescription is made up of one or more acupoint groups. Each group consists of acupoints that are influential points, empirical points, or local points. There are 39 formulae (combinations) of acupoints, which are practical and finely selected.
Scalp acupuncture	SAT	It is a contemporary acupuncture technique integrating traditional Chinese needling methods with Western medical knowledge of representative areas of the cerebral cortex. It has been proven to be a most effective technique for treating acute and chronic central nervous system disorders. Scalp acupuncture often produces remarkable results with just a few needles and usually brings about immediate improvement, sometimes taking only several seconds to a minute.
Warm-needle acupuncture	WNA	A method combining acupuncture with moxibustion, a moxa column is added to the end of the needle, ignites it, and the heat is transmitted through the needle to the body after being ignited to treat diseases.
Acupoint catgut embedding	ACE	An involves weekly infixing of surgical chromic catgut sutures into the subcutaneous tissue of acupoints located at the abdomen, extremities, and the back with a specialized needle under aseptic precautions.
Bee venom acupuncture	BVA	It is a kind of herbal acupuncture taking advantage of diluted bee venom instead of distilled herbal decoction.
Needle knife acupuncture	NKA	It is a closed minimally invasive surgery, which integrates the acupuncture in modern Chinese medicine with the knife in surgery. It can restore the biomechanical balance, release the soft tissue, improve the internal microcirculation, reduce the internal pressure of bone, alleviate the inflammatory reaction, and regulate the meridians.
Moxibustion	Moxa	A method in which a moxa herb is burned above the skin or on the acupuncture points. It can be used as a cone stick, loose herb, or applied at the end of the acupuncture needles. The purpose of moxibustion is to apply heat to the acupuncture points to alleviate symptoms.
Chinese Herbal medicine	CHM	It is part of a larger healing system called traditional Chinese medicine (TCM). Herbs are prescribed to restore energy balance to the opposing forces of energy - Yin and Yang - that run through invisible channels in the body.
Herbal fumigation	HF	A branch of Chinese medicine external treatment, also called traditional Chinese medicine steaming therapy, traditional Chinese medicine steam bath therapy. A combination of chemical and physical therapy with hot medicine steam as the therapeutic factor.
Herbal socking	HS	Herbal soaking method is to boil a suitable Chinese medicine formula into Chinese medicine water and soak the feet. With the help of hot water, the effective Chinese medicine ingredients penetrate into the skin, are absorbed by the capillaries of the feet, and enter the human blood circulation system to achieve.
Taichi	TC	A kind of traditional Chinese exercise that share the common Chinese medicine theory and place an emphasis on a combination of physical exercise with mental focus; interaction between physical postures and movements, meditative mind, musculoskeletal relaxation and breathing techniques in a harmonious manner.
Baduanjin	BDJ	A kind of traditional Chinese qigong that has been low-intensity aerobic exercised for a long time in China as a mind-body exercise.
Daoyin	DY	A kind of traditional Korean medicine exercise, it is divided into an exercise method in which the patient performs active exercise together with breathing and an exercise method in which the operator passively treats the patient.
Tuina	TN	Tuina is a kind of external therapy belonging to traditional Chinese medicine. Tuina massage stimulates the flow of qi to promote balance and harmony within the body using many of the same principles of acupuncture.

TCM: Traditional Chinese medicine

Supplementary Table 3. Assessment of all included systematic reviews using AMSTAR 2.

Study ID	1	2	3	4	5	6	7	8	9	10	11	12	13	14	15	16	Rating overall confidence*
Zhou (2020)(12)	Yes	No	Yes	Yes	Yes	Yes	No	Yes	Yes	Yes	Yes	Yes	Yes	Yes	No	Yes	Moderate
Zhan (2017)(13)	Yes	No	Yes	Yes	Yes	Yes	No	Yes	Yes	Yes	No	Yes	Yes	No	Yes	No	Low
Xiong (2016)(14)	Yes	No	No	Yes	No	Yes	No	Yes	Yes	Yes	Yes	No	Yes	No	No	No	Critically low
Shen (2020)(15)	Yes	No	Yes	Yes	Yes	No	No	Yes	Yes	Yes	Yes	No	Yes	No	No	No	Low
Lin (2015)(16)	Yes	No	No	Yes	Yes	Yes	No	Yes	Yes	Yes	Yes	No	Yes	No	No	No	Critically low
Wei (2019)(17)	Yes	No	Yes	Yes	Yes	Yes	No	Yes	Yes	Yes	Yes	Yes	Yes	Yes	Yes	No	Low
Lim (2015)(18)	Yes	No	No	Yes	No	Yes	No	Yes	Yes	Yes	Yes	No	Yes	No	No	No	Critically low
Oh (2020)(19)	Yes	No	Yes	Yes	Yes	Yes	Yes	Yes	Yes	No	Yes	Yes	Yes	No	No	No	Low
Liu (2019)(20)	Yes	No	Yes	Yes	Yes	Yes	No	Yes	Yes	Yes	Yes	Yes	Yes	No	No	Yes	Low
Wang (2019)(21)	Yes	No	Yes	Yes	Yes	Yes	No	Yes	Yes	No	Yes	Yes	Yes	Yes	No	No	Low
Li (2016)(22)	Yes	No	Yes	Yes	Yes	Yes	No	Yes	Yes	No	No	Yes	Yes	No	No	No	Critically low
An (2017)(23)	Yes	No	Yes	Yes	Yes	No	No	Yes	Yes	No	Yes	Yes	Yes	No	No	No	Critically low
Wu (2018)(24)	Yes	No	Yes	Yes	Yes	Yes	No	Yes	Yes	Yes	Yes	Yes	Yes	Yes	Yes	No	Low
Hou (2021)(25)	Yes	No	Yes	Yes	Yes	Yes	No	Yes	Yes	No	Yes	Yes	Yes	Yes	No	No	Low
Lin (2017)(26)	Yes	No	Yes	Yes	Yes	Yes	No	Yes	Yes	No	No	Yes	Yes	No	No	No	Critically low
Zheng (2020)(27)	Yes	No	Yes	Yes	No	Yes	No	Yes	Yes	No	Yes	Yes	Yes	Yes	Yes	No	Low
Wang (2020)(28)	Yes	No	Yes	Yes	Yes	Yes	No	Yes	Yes	No	Yes	Yes	Yes	Yes	No	No	Low
Guo (2015)(29)	Yes	No	Yes	Yes	No	No	No	Yes	Yes	Yes	No	Yes	Yes	No	Yes	No	Critically low
Qi (2018)(30)	Yes	No	Yes	Yes	Yes	Yes	No	Yes	Yes	No	Yes	Yes	Yes	Yes	No	No	Low
Zhang (2016)(31)	Yes	No	Yes	Yes	Yes	No	No	Yes	Yes	No	Yes	Yes	Yes	No	Yes	No	Low
Zhan (2016)(32)	Yes	No	Yes	Yes	Yes	No	No	Yes	Yes	Yes	Yes	Yes	Yes	No	Yes	No	Critically low
Lyu (2018)(33)	Yes	No	Yes	Yes	Yes	No	No	Yes	Yes	No	Yes	Yes	Yes	No	Yes	No	Low
Zou (2018)(34)	Yes	No	Yes	Yes	Yes	Yes	No	Yes	Yes	Yes	Yes	Yes	Yes	Yes	No	Yes	Moderate
Zheng (2016)(35)	Yes	No	Yes	Yes	Yes	Yes	No	Yes	Yes	Yes	No	No	Yes	Yes	No	No	Low
Zhan (2018)(36)	Yes	Yes	Yes	Yes	Yes	Yes	No	Yes	Yes	Yes	Yes	Yes	Yes	Yes	Yes	Yes	Moderate
Ye (2017)(37)	Yes	No	Yes	No	Yes	No	No	Yes	Yes	No	No	Yes	Yes	No	No	No	Critically low
Qiu (2021)(38)	Yes	No	Yes	No	Yes	No	No	Yes	Yes	No	No	Yes	Yes	No	No	Yes	Moderate
Yang (2018)(39)	Yes	No	Yes	No	Yes	No	No	Yes	Yes	No	No	Yes	Yes	No	No	No	Critically low
Cai (2019)(40)	Yes	No	Yes	Yes	Yes	Yes	No	Yes	Yes	Yes	No	No	Yes	Yes	No	Yes	Low
Yan (2015)(41)	Yes	No	Yes	No	Yes	No	No	Yes	Yes	No	No	Yes	Yes	No	No	No	Critically low
Fan (2020)(42)	Yes	No	Yes	Yes	Yes	No	No	Yes	Yes	Yes	Yes	Yes	Yes	Yes	Yes	Yes	Moderate
Li (2021)(43)	Yes	No	Yes	Yes	Yes	Yes	No	Yes	Yes	No	Yes	Yes	Yes	Yes	No	No	Low
You (2020)(44)	Yes	No	Yes	Yes	Yes	Yes	No	Yes	Yes	Yes	No	Yes	Yes	Yes	No	No	Low
Xie (2020)(45)	Yes	No	Yes	Yes	Yes	Yes	No	Yes	Yes	Yes	Yes	Yes	Yes	No	No	No	Low
Yu (2019)(46)	Yes	No	Yes	No	Yes	No	No	Yes	Yes	No	Yes	Yes	Yes	No	No	No	Critically low
Wen (2020)(47)	Yes	No	Yes	Yes	Yes	No	No	Yes	Yes	No	Yes	Yes	Yes	Yes	No	No	Low
Chen (2016)(48)	Yes	No	Yes	No	Yes	No	No	Yes	Yes	No	Yes	Yes	Yes	No	No	No	Critically low
Ma (2017)(49)	Yes	No	Yes	Yes	Yes	No	No	Yes	Yes	No	No	Yes	Yes	No	No	No	Critically low
Fan (2016)(50)	Yes	No	Yes	No	Yes	No	No	Yes	Yes	No	No	Yes	Yes	No	No	No	Critically low
Chen (2020)(51)	Yes	No	Yes	Yes	Yes	Yes	No	Yes	Yes	Yes	Yes	Yes	Yes	Yes	Yes	No	Moderate
Liu (2017)(52)	Yes	No	Yes	No	Yes	Yes	No	Yes	Yes	Yes	Yes	No	Yes	No	Yes	No	Critically low
Zhang (2020)(53)	Yes	No	Yes	Yes	Yes	Yes	No	Yes	Yes	Yes	No	Yes	Yes	Yes	Yes	No	Low
Gou (2019)(54)	Yes	No	Yes	Yes	Yes	Yes	No	Yes	Yes	Yes	No	Yes	Yes	No	Yes	No	Low
Ji (2020)(55)	Yes	No	Yes	Yes	Yes	Yes	No	Yes	Yes	Yes	Yes	Yes	Yes	Yes	Yes	No	Moderate
Lee (2019)(56)	Yes	No	Yes	Yes	Yes	Yes	No	Yes	Yes	Yes	Yes	Yes	Yes	Yes	No	No	Low
Wang (2018)(57)	Yes	No	Yes	Yes	Yes	Yes	No	Yes	Yes	Yes	Yes	Yes	Yes	Yes	No	No	Low
Lin (2015)(58)	Yes	No	Yes	Yes	Yes	Yes	No	Yes	Yes	Yes	Yes	Yes	Yes	Yes	Yes	Yes	Moderate
Bu (2017)(59)	Yes	No	Yes	No	Yes	No	No	Yes	Yes	No	No	Yes	Yes	No	No	No	Critically low

*AMSTAR 2: A MeaSurement Tool to Assess systematic Reviews 2; AMSTAR2 was used to critically appraise the reporting quality of each included SR. The overall confidence of each SR was graded as “high” (no or non-critical weakness in all items), “moderate” (more than one non-critical weakness among all the items), “low” (one critical flaw with or without non-critical weakness), or “critically low” (more than one critical flaw with or without non-critical weakness).

1. Components of PICO; 2. Established prior to the conduct of the review; 3. Explain their selection of the study designs; 4. Comprehensive search; 5. Duplicate selection; 6. Duplicate extraction; 7. List of excluded studies and justify the exclusions; 8. Describe the included studies in adequate detail; 9. Use a satisfactory technique for assessing the risk of bias (RoB); 10. Report on the sources of funding; 11. Use appropriate methods for statistical combination of results; 12. Assess the potential impact of RoB in individual studies on the results; 13. Account for RoB in individual studies when interpreting/discussing the results of the review; 14. Provide a satisfactory explanation for, and discussion of, any heterogeneity observed in the results of the review; 15. Publication bias assessed; 16. Include conflict of interest

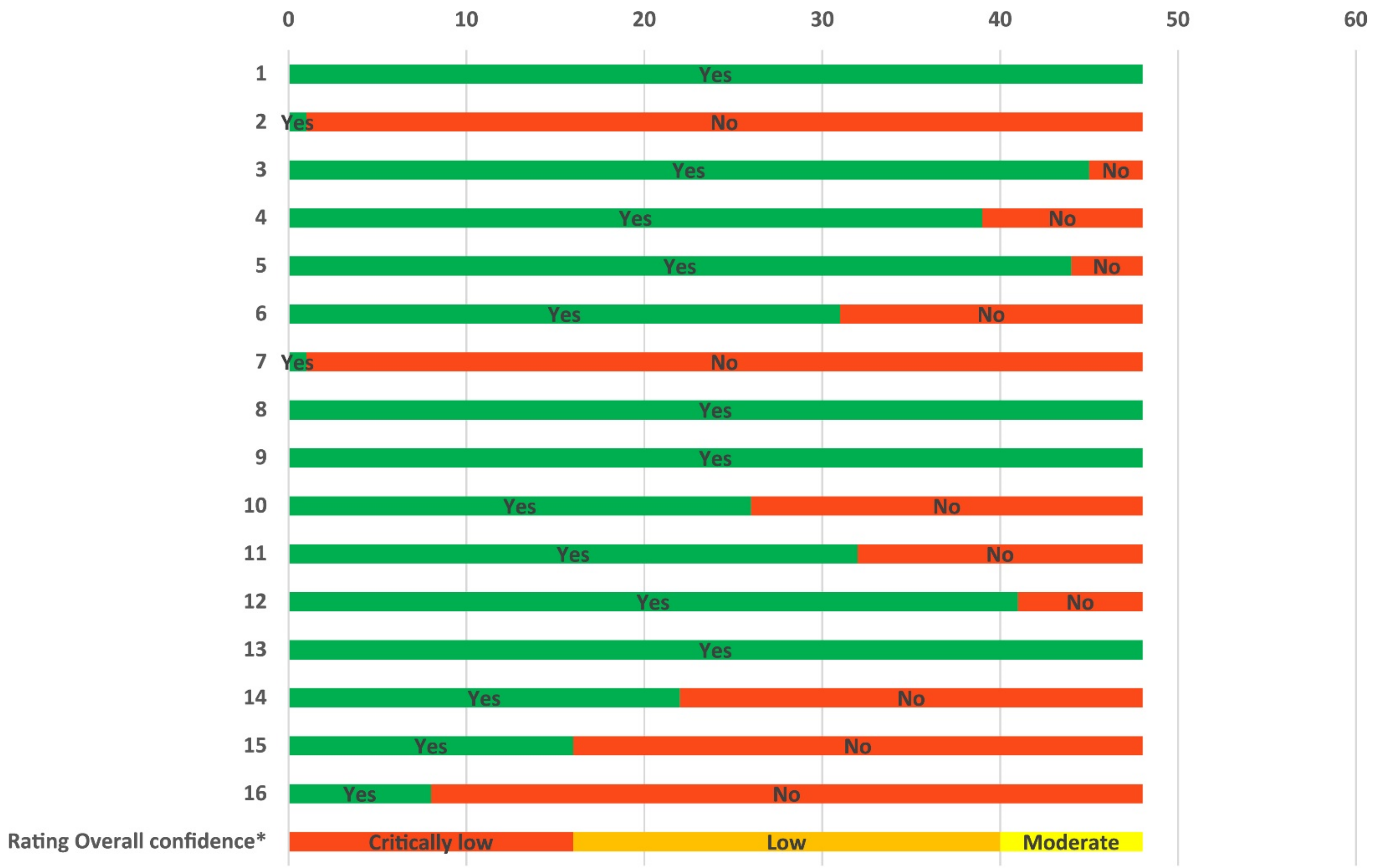

Supplementary Figure 1. Assessment of all included systematic reviews using AMSTAR 2.

1. Components of PICO; 2. Established prior to the conduct of the review; 3. Explain their selection of the study designs; 4. Comprehensive search; 5. Duplicate selection; 6. Duplicate extraction; 7. List of excluded studies and justify the exclusions; 8. Describe the included studies in adequate detail; 9. Use a satisfactory technique for assessing the risk of bias (RoB); 10. Report on the sources of funding; 11. Use appropriate methods for statistical combination of results; 12. Assess the potential impact of RoB in individual studies on the results; 13. Account for RoB in individual studies when interpreting/discussing the results of the review; 14. Provide a satisfactory explanation for, and discussion of, any heterogeneity observed in the results of the review; 15. Publication bias assessed; 16. Include conflict of interest
